# Fifteen essential science advances needed for effective restoration of the world's forest landscapes

**DOI:** 10.1098/rstb.2021.0065

**Published:** 2023-01-02

**Authors:** Andrew R. Marshall, Catherine E. Waite, Marion Pfeifer, Lindsay F. Banin, Sarobidy Rakotonarivo, Susan Chomba, John Herbohn, Donald A. Gilmour, Mark Brown, Robin L. Chazdon

**Affiliations:** ^1^ Forest Research Institute, University of the Sunshine Coast, QLD 4556, Australia; ^2^ Department of Environment and Geography, University of York, York YO10 5DD, UK; ^3^ Reforest Africa, Mang'ula, Tanzania; ^4^ Flamingo Land Ltd, Kirby Misperton, North Yorkshire YO17 6UX, UK; ^5^ School of Natural and Environmental Sciences, Newcastle University, Newcastle upon Tyne NE1 7RU, UK; ^6^ UK Centre for Ecology & Hydrology, Library Avenue, Bailrigg, Lancaster LA1 4AP, UK; ^7^ École Supérieure des Sciences Agronomiques, Université d'Antananarivo, BP 566 Antananarivo, Madagascar; ^8^ World Resources Institute, Nairobi, Kenya

**Keywords:** forest, forest landscape restoration, restoration, strategic planning, socio-ecological systems, United Nations

## Abstract

There has never been a more pressing and opportune time for science and practice to collaborate towards restoration of the world's forests. Multiple uncertainties remain for achieving successful, long-term forest landscape restoration (FLR). In this article, we use expert knowledge and literature review to identify knowledge gaps that need closing to advance restoration practice, as an introduction to a landmark theme issue on FLR and the UN Decade on Ecosystem Restoration. Aligned with an Adaptive Management Cycle for FLR, we identify 15 essential science advances required to facilitate FLR success for nature and people. They highlight that the greatest science challenges lie in the conceptualization, planning and assessment stages of restoration, which require an evidence base for why, where and how to restore, at realistic scales. FLR and underlying sciences are complex, requiring spatially explicit approaches across disciplines and sectors, considering multiple objectives, drivers and trade-offs critical for decision-making and financing. The developing tropics are a priority region, where scientists must work with stakeholders across the Adaptive Management Cycle. Clearly communicated scientific evidence for action at the outset of restoration planning will enable donors, decision makers and implementers to develop informed objectives, realistic targets and processes for accountability. This article paves the way for 19 further articles in this theme issue, with author contributions from across the world.

This article is part of the theme issue ‘Understanding forest landscape restoration: reinforcing scientific foundations for the UN Decade on Ecosystem Restoration’.

## 1. Introduction

The world's forests face unprecedented challenges and an uncertain future [1]. Conservation and restoration of forests are at the centre of global efforts to mitigate climate change and prevent mass extinctions of biodiversity. Despite considerable ambition to halt deforestation and to restore forest landscapes, progress has been slow and difficult to measure, and forest fragmentation, loss and degradation continue [1,2]. Social aspects of degradation and recovery continue to be overlooked [3,4], compromising long-term conservation success and exacerbating injustice, food insecurity and displacement [5]. Forest landscape restoration (FLR) has emerged as a promising approach to reverse degradation, aiming to improve both livelihoods and environmental conditions with active participation of local communities [6]. FLR confronts many challenges [7] but lacks a comprehensive evidence base for guiding effective restoration efforts in different forest biomes and socio-political contexts.

**Figure 1.**
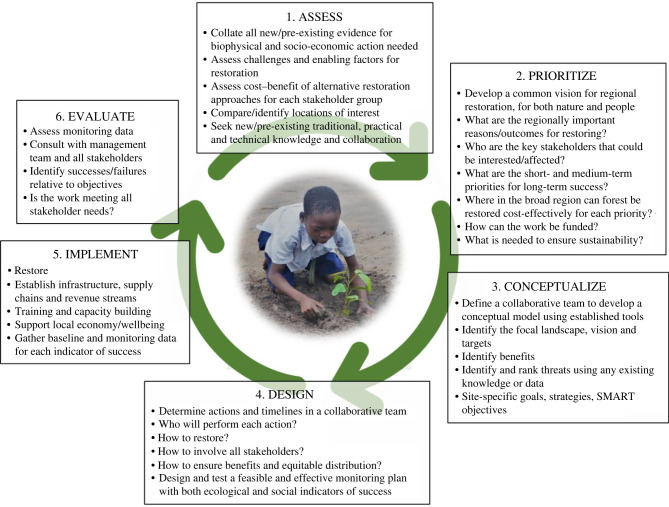
An Adaptive Management Cycle for Forest Landscape Restoration. Adapted from the Conservation Standards [8]. Photo credit (with permission): Revocatus Laurian, Reforest Africa. (Online version in colour.)

This article paves the way for a theme issue of 19 further articles, collectively highlighting and expanding scientific understanding relevant for FLR. It was inspired by the multiple uncertainties that remain in forest restoration science, recent debates and misconceptions in the scientific literature, and by the United Nations (UN) Decade on Ecosystem Restoration. To emphasize the relevance of restoration science for practice, we structure the theme issue around a restoration Adaptive Management Cycle (AMC; figure 1), a widely advocated procedure for ecosystem management [9–12]. The principle behind restoration adaptive management is that projects will benefit from ongoing monitoring and evaluation to determine success and to revise objectives and actions based on emerging knowledge and experience. The theme issue begins with seven articles identifying pathways and constraints for forest restoration that may require consideration at the project conception stage. Subsequently, four articles address restoration planning and evaluation, and six articles address techniques for implementing forest restoration. Our introductory article also provides a scientific overview for the theme issue, while two prefaces highlight the academic and policy priorities for science in the Decade on Ecosystem Restoration.

There has never been a more important time to deliver the scientific foundations for effective and long-lasting impacts of forest restoration that meets the needs and priorities of different stakeholders. The Decade on Ecosystem Restoration aims to mobilize action, unlock new sources of financial support and create a global restoration movement involving all sectors of society. Science plays a crucial role in this ambition to achieve global restoration targets, and opportunities for engaging scientists from a broad range of disciplines are emerging through various platforms and partner organizations [13]. Making restoration science more relevant, improving communication and aligning with other research fields have the potential to substantially improve the speed of uptake of restoration science by restoration practitioners [14]. In support of this, our definition of ‘restoration’ aligns with the United Nations Environment Programme (UNEP) definition of ecosystem restoration, ‘Ecosystem restoration is the process of halting and reversing degradation, resulting in improved ecosystem services and recovered biodiversity. Ecosystem restoration encompasses a wide continuum of practices, depending on local conditions and societal choice’.

To be effective in delivering benefits to all stakeholders, the International Union for Conservation of Nature (IUCN)/UNEP team overseeing the Decade on Ecosystem Restoration Science Task Force emphasize in this theme issue the need to focus critically on evidence-based policy design and decision-making, action and monitoring [13]. However, the UN-defined knowledge gaps for the restoration process are quite broad, and there has been limited systematic attempt to identify priority science advances [15] or indicators of success [16], despite reasonable appreciation of ongoing challenges in FLR [10,17,18].

Here we propose a list of essential science advances that form a research agenda for science to effectively facilitate global FLR success for both nature and people. We begin by identifying components of the AMC where science can most likely provide support for restoring forest landscapes. We then introduce 15 science advances that address these components, developed from expert opinion, submissions to the theme issue, and knowledge gaps identified by the UN. We consider current knowledge gaps within each proposed advance, using examples from the theme issue and broader literature. Subsequently, we outline our suggestions to prioritize future directions for science and practice.

## 2. Essential science advances

### (a) Our approach

**Figure 2.**
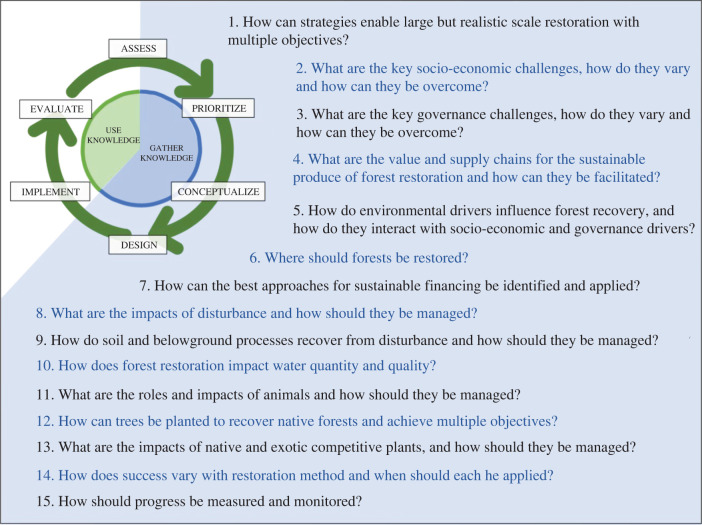
What science advances would best help the AMC for forest landscape restoration? Fifteen essential science questions are shown in the approximate order that they become important during knowledge-gathering. Advances towards questions 1–7 would facilitate planning (Assess, Prioritize and Conceptualize), while advances towards questions 8–15 would facilitate the Design stage, for later use in the implementation stages (Implement and Evaluate). (Online version in colour.)

To develop an AMC for restoring forest landscapes (figure 1), the guest editors of this theme issue adapted a conservation cycle from the widely adopted Conservation Standards [8]. Collectively, the team had 125 years of relevant experience, working across multiple sectors in more than 30 countries, across all forested continents, hence bringing a diverse set of knowledge frameworks and credentials. By considering each step of the AMC, the team used their expert opinion and literature review to identify critical knowledge gaps that require scientific input to improve AMC decision-making and implementation. A provisional list of these essential advances was then circulated to more than 100 experts in forest restoration science and practice, and two anonymous reviewers, as a result of which we modified and finalized the list (figure 2; electronic supplementary material, S1). All but one of the 15 advances are addressed in more detail in the theme issue (table 1).

**Table 1.** List of research articles making progress towards 15 essential science advances for effective restoration of the world's forest landscapes within this theme issue.

Essential advance	Contributing articles (first author name)
*Advances for FLR planning*	
1. Strategic planning	Gnacadja; Pfeifer; Wills
2. Socio-economic challenges	Herbohn; Loveridge; Pfeifer; Tedesco; Wills
3. Governance challenges	Gnacadja; Loveridge; Tedesco; Wills
4. Value and supply chains	Herbohn; Loveridge
5. Environmental versus other drivers	Banin; König; Pfeifer; Stas
6. Where to restore	Lewis K; Pfeifer; Wills
7. Sustainable financing	Herbohn; Loveridge; Tedesco; Wills
*Advances for FLR implementation*	
8. Disturbance impact/management	König; Lindenmayer; Pfeifer; Stas; Wills
9. Soil and below-ground processes	König; van der Sande; Werden
10. Impacts on water	–
11. Animal impacts and roles	Estrada-Villegas; Pfeifer
12. Tree-planting	Banin; König; Kulikowski; Matos; Pfeifer; Stas; Werden; Wills
13. Competitive plants	Banin; Kulikowski; Wills
14. Restoration methods	Banin; Elliott; Kulikowski; Matos; Wills
15. Monitoring outcomes	Banin; Chazdon; Estrada-Villegas; Gnacadja; Herbohn; Lewis SL; Loveridge; Pfeifer

### (b) Advances for forest landscape restoration planning

Advances 1–7 are most relevant to the planning stages of FLR, which require extensive collation of information through document review, stakeholder engagement and discussion among project team members and collaborators. Science has often been relatively peripheral to this process and yet has huge potential to improve procedures through evidence-based assessment of site selection, protocols, threats, challenges and financial sustainability.

#### (i) Advance 1. How can strategies enable large- but realistic-scale restoration with multiple objectives?

Strategic planning in FLR is the process of setting a vision, goals, multiple objectives and actions, for a region identified for restoration, considering both the desired future status of forests and the people that interact with them (figure 1—Conceptualize and Design stages). To achieve these, FLR practices must be tailored to region-specific socio-economic, governance and biophysical contexts, because some approaches may not work in all contexts, demanding flexible applications and adherence to principles for good practice [7]. This process is more effective when multiple spatially based aspects are integrated to identify priority interventions, e.g. socio-economic, ecological and climate, and their trade-offs and synergies [19,20] and when science and local place-based knowledge are combined [21,22]. Global and multi-national assessments of restoration potential have inspired scientific advancement, drawing media and political attention [23]. However, they lack regional-level details and engagement ([24,25]; Advance 2) and run the risk of creating unrealistic targets that are less likely to succeed in the long term and exacerbate injustice, food insecurity and displacement [26].

Among various approaches for developing restoration strategies [27], the Restoration Opportunities Assessment Methodology (ROAM) is widely used for national and subnational FLR planning based on locally relevant spatial data and diverse stakeholder input [28]. Conservation planning tools also have potential for application in the restoration sector, e.g. the Conservation Standards [8]. Threat reduction assessment, results chains, theories of change and indicator selection are all crucial elements of restoration and conservation planning that can benefit from scientific oversight. The spatial multi-criteria decision-making methods embedded into some of these tools are especially important for land-use planning and collaborative management ([29,30]; Advances 2 and 3). Different tools evaluate trade-offs and synergies of these criteria for spatially complex decision-making processes [24].

For science to facilitate multiple restoration objectives, it is essential that strategic, spatial assessments of trade-offs and synergies are completed at scales relevant for planning. Our policy preface to this theme issue emphasizes that these assessments must consider all relevant development priorities to find cross-sector solutions [13]. New research in the theme issue uses a systems approach to jointly consider the biodiversity, human wellbeing and food security implications of restoration in forest–agricultural landscapes using multiple criteria, pathways and outcomes [31]. Further research in the theme issue uses spatial data from a large landscape with multiple land uses, to demonstrate that the choice of criteria, data layers and timeframes used for evaluation strongly impacts the results of multi-criteria decision-making methods [32].

#### (ii) Advance 2. What are the key socio-economic challenges, how do they vary and how can they be overcome?

At least 12% of the population in low-income countries live on forest restoration opportunity land [6] and 1.2 billion people are directly dependent on nature for everyday life [33]. While forest restoration can produce ecosystem services for these communities, it can also generate disservices (e.g. negative health impacts from pathogens, crop damage by wildlife pests). These, in turn, may play an important role in stakeholder decision-making [34] and may lead to misconceptions and opportunity costs, especially reduced agricultural land [35]. Restoration planning, prioritization modelling and mapping can therefore have substantial equity and justice implications [5]. And yet, global priority areas for ecosystem restoration identified from biodiversity, climate and cost, included no socio-cultural and livelihood objectives [36,37]. Moreover, there is uncertainty regarding how this could be done at all at such a large scale [38]. To compound this, as highlighted in this theme issue, scientific findings from socio-economic research only rarely inform the political and restoration investment decisions of governments and development partners [13]. A further challenge for FLR is that the geopolitics of restoration investments can be driven by objectives unrelated to maximizing biodiversity or wellbeing outcomes [4].

Improved data collection (including social alongside ecological variables), sharing and analyses are needed to design more effective, fair and equitable FLR processes [35,39,40]. Research is needed into the mechanisms by which ecosystem services and disservices may accrue to different stakeholders following successful FLR [41] and increasing engagement with local communities and marginalized groups, e.g. through participatory approaches. These considerations will allow the estimation of more realistic opportunity costs [42] and better understanding of motivations, knowledge, challenges and benefits across different disciplinary stakeholders, especially women and others with limited power and agency [43]. Testing and promoting the uptake of existing tools, such as participatory scenario building and modelling to incorporate perspectives and value systems of different stakeholders, can help to navigate uncertainties surrounding future trajectories of landscapes. The people, authorities and organizations interacting with, and affected by, forest restoration are diverse, with different roles and scales of operation and each deriving benefits or disbenefits from the restoration, e.g. international donors, national forestry agencies and local landowners. Approaches for gathering information from these multiple stakeholders are therefore both context- and stakeholder-specific.

Projects often assume win–win narratives for biodiversity and ecosystem services [44]. In this theme issue, we see how human wellbeing in regions of forest restoration can benefit from good governance (Advance 3), crop yields and access to forest resources [45], but with conflicting benefits and costs in relation to biodiversity [31]. Further new research in the theme issue shows that around half of all incentive schemes have resulted in perceived win–wins, but that adverse outcomes are more commonly socio-economic than ecological [46]. Emerging theory in the theme issue also highlights the potential for socio-economic evaluation to identify tipping points in community capacity for restoration [47].

#### (iii) Advance 3. What are the key governance challenges, how do they vary and how can they be overcome?

The governance of restoration sites often determines success, e.g. land tenure, management systems, institutions, collaborations and policies. The diverse objectives across FLR landscapes (Advance 1) require diverse and participatory governance approaches [48]. However, many restoration projects still take a top-down approach, where decision-making often overlooks local communities [49] or prioritizes outside experts or external actors [50], consequently worsening social and potentially also environmental outcomes [51]. Power imbalances and conflicting interests between funders, government, project implementers and local communities shape who influences restoration priorities and approaches [17]. FLR that does not secure local communities' consent and engagement, nor address potential negative social impacts, might lead to forced displacement, unjust climate mitigation and costly monitoring and regulation to prevent illegal, but often legitimate, activities [6].

Many restoration initiatives begin with mapping forest restoration opportunities without examining how such lands are used, contested or governed [52]. Land tenure, in the developing tropics as elsewhere, can be complex, dynamic and contested, while customary and statutory land rights are often disconnected [53]. In instances where tenure is unclear or contested, restoration without prior consent may be akin to conservation land grabs [17,54]. Secure tenure to land and trees can incentivize community support, giving stronger negotiation power and ability to influence restoration planning and outcomes [52], while those with insecure tenure or renting land will be unlikely to benefit. And yet, land tenure does not always affect farmer decisions and is highly dependent on other aspects of wellbeing [55].

Moving forward, case studies are needed to identify sustainable and equitable governance approaches, and the influence of varying governance on FLR success. There is little published information on the wellbeing and ecological impacts of varying restoration rules, regulatory processes, incentive schemes, types and number of organizations, and between different forms of protected area and private land tenure. In this theme issue, we see that governance challenges can be closely aligned to socio-economic challenges (Advance 2) because equitable forest governance promotes human wellbeing, as highlighted for a forest certification scheme [45]. Similarly, a literature review in the theme issue demonstrates that good governance is crucial for the effectiveness of forest restoration incentive schemes [46]. Also in this theme issue, we see that low restoration costs within protected areas highlight a temptation for donors and implementers to follow the simplest paths for achieving restoration targets, overlooking community restoration needs on public or private land [32].

#### (iv) Advance 4. What are the value and supply chains for the sustainable produce of forest restoration and how can they be facilitated?

Natural forests are mostly less economically valuable to local economies than alternative land uses, and hence local opportunity costs of forest restoration often outweigh local benefits (Advances 2 and 3). With better understanding of how native timber and other forest and agroforest products can contribute more directly and effectively to local economies, forests will likely become more profitable and more locally desirable [56]. There needs to be both a clear definition and understanding of which governance and interventions can deliver value to the local community and what the community capacity is to realize these values [6,41]. In relation to sustainable forest product values, limitations in infrastructure or country border crossing significantly reduce or eliminate access to high-value international markets [57]. Similarly with new and emerging non-timber values like carbon and biodiversity certification, any access to international markets will require reliable monitoring and reporting for any monetary value from outside the local community.

At the community level, communities at different points on the community capacity curve (see [47] in this theme issue) have different capabilities (e.g. human, physical and financial capitals) to engage in value and supply chains. Any initiatives must recognize community capacity and either identify opportunities that fit existing capacity or develop the appropriate community capacity. Critical in the research is the development of frameworks to identify local community capacity, local value proposition in the timber and non-timber space, and standardized approaches to connect the two through long-term forest restoration objectives, including the identification of, and strategies to fill, key gaps. Research to address the profitability of entrepreneurial small-scale land managers needs to provide systems and tools to improve market access with more accessible processes to international certification alongside the development of diverse local markets for timber and non-timber products [58]. However, research in this theme issue also highlights that revenue may take time to accrue, emphasizing its combined importance with other governance and socio-economic factors [45].

#### (v) Advance 5. How do environmental drivers influence forest recovery, and how do they interact with socio-economic and governance drivers?

Improved understanding of forest responses to varying climate, soil and topography are essential for making accurate projections of recovery and restoration across landscapes, and hence also for identifying restoration methods and costs. Available evidence suggests that recovery of forests from disturbance is highly variable, ranging from tens to greater than 1000 years for forest biomass [59,60]. Forest recovery from heavy disturbance can be rapid where water and temperature conditions are favourable [61–64]. However, the available data and models of secondary forest recovery are heavily biased towards the neotropics and mostly lack consideration of human factors (Advances 2 and 3), restoration interventions (Advances 12 and 14), and stalled or time-lagged recovery resulting from non-environmental feedbacks, e.g. competing vegetation (Advances 13 and 15).

Assessing and integrating environmental, socio-economic and governance drivers of restoration success within a particular socio-ecological context are challenging. For example, drivers can be either directly or indirectly influential, and there are spatial scales of variation in driver–response relationships, interactions and feedbacks. Legislative policies and land rights often apply to entire countries or subregions, whereas environmental conditions that promote restoration can vary within a single property, watershed or jurisdiction. A recent path-analysis revealed that landscape-scale biodiversity recovery during natural forest regeneration was directly positively associated with nearby forest cover, and negatively associated with urbanization, suggesting that favourable biophysical conditions along with low socio-economic pressures are crucially important [65]. These land-use factors mediated indirect associations with a wide range of social and ecological factors, including economic opportunities, social needs and ecological and biophysical conditions. A landscape-scale meta-analysis further found that forest biodiversity recovery could be predicted by 14 out of 45 socio-environmental factors, relating to human demography, land use, disturbances, productivity, water, topography and soil [66].

When environmental conditions are challenging for restoration, financial costs of interventions are likely to be higher, and specialized training of practitioners will be necessary, such as following mining operations [67]. Therefore, more work is needed to understand the relative importance of the multiple drivers of restoration success, and how they interact. In this theme issue, wind damage in Vietnam is shown to be more detrimental to forest recovery where past disturbance and land-use change have been greatest [68], while planted seedling survival across Southeast Asian sites was highly variable, but particularly connected to habitat condition at time of planting [69]. Similarly, new data in the theme issue from Brazil show an increase in forest restoration success with soil quality and proximity to forests [70].

#### (vi) Advance 6. Where should forests be restored?

Effective determination of where to restore is an essential component of FLR strategic planning (Advance 1). Armed with information from the preceding Advances, a restoration project team should be in a reasonable place to decide where to restore forests to best balance trade-offs for both nature and people, at appropriate scales and timeframes. Spatial priority planning is used to deliver on biodiversity and carbon sequestration targets from landscape [71,72] to global [37] scales. Spatial prioritization approaches, e.g. the widely used decision-support tool Marxan [73], can enable the efficient allocation of resources to areas identified as important for different outcomes [74], helping management to decide on locations that minimize impact on stakeholders and maximize co-benefits. Their use for spatial restoration planning requires evidence on the appropriate data for these tools and their interrelationships across scales.

However, research into determining where to restore, and appropriate spatial scales for decision making, is limited, especially when aiming to incorporate social indicators (Advance 2). And yet, this research is crucial for balancing environmental suitability and opportunity and implementation costs. Identifying the importance of forests relative to other ecosystems is also a vital but overlooked aspect of FLR spatial decision making. Forests are not always the climax ecosystem, or the only ecosystem in need of restoration, and ecosystems that were not formerly forests should not be managed towards a forested state [75]. Thus, determining where to restore forests should also involve determining where not to restore them. In this theme issue, ecosystem-specific spatial restoration assessment in the Brazilian Cerrado reveals high potential for both active and passive restoration [76]. This same work also highlights that locations identified as hotspot areas for restoration using global datasets do not always overlap with areas prioritized by national restoration commitments. Similar data from Tanzania are also coupled with logistical data to reveal greater regional potential than previously inferred by global assessments [32]. Further research in the theme issue shows how alternative positioning of forest restoration corridors can affect levels of wellbeing and human–wildlife conflict [31].

#### (vii) Advance 7. How can the best approaches for sustainable financing be identified and applied?

Restoration can be financed in many different ways, and a recent framework for financing FLR identifies both public and private sources [77]. An important area for research is to identify the types of funding best suited to alternative restoration projects, e.g. how can the most appropriate finance options be pre-determined using socio-economic, infrastructure, policy, biological and environmental features of a site/region? Much has been made of the potential for private sector investment and carbon credit schemes in restoration (e.g. [78]), but knowledge is limited regarding their sustainability and capacity to enhance wellbeing, particularly in socially complex tropical landscapes [11]. Biodiversity credits also offer potential, but a fundamental question remains as to how these credits can be quantified in a form suitable for trading [79].

Critically, most projects receive short-term funding, while restoration is a long-term venture; hence targets and financial strategies need to consider both donor and sustainable timeframes. Currently, funding is typically provided only for the restoration intervention to be implemented, as opposed to ongoing maintenance, and the various infrastructure requirements around a project. The real cost of restoration is much greater than this, but little information is available as to what these real costs are to ensure long-term survival of the tree planted. There are also critical information gaps (i.e. robust and consistent data) concerning the relative costs and benefits of restoration [42] and hence also procedures for evaluating and mitigating risk for donors.

In this theme issue, forest certification and restoration incentive schemes are highlighted as positive examples for sustainable financing, but with no standard solution for meeting multiple objectives [45,46]. A spatial assessment of restoration cost in the theme issue also shows how relative outcomes for different approaches differ with alternative funding timeframes and highlights that real community restoration costs need more research, and more thorough documenting [32]. In the theme issue we also see that, for many developing tropical countries, communities with greater financial and social capitals need less financial support [47].

### (c) Advances for forest landscape restoration implementation and evaluation

Advances 8–15 are most relevant to deciding between alternative activities for restoring forest landscapes, for addressing socio-economic challenges and threats and for measuring and monitoring progress. Science is needed to provide evidence for the strengths and weaknesses of different activities, and under what circumstance each is most appropriate.

#### (i) Advance 8. What are the impacts of disturbance and how should they be managed?

Many forms of disturbance are increasing in scale and scope globally [80]. Restoration efforts can be delayed and compromised by any past or ongoing disturbance that disrupts the ecosystem, affecting resources, species interactions or the physical environment, e.g. fire, logging, mining, cyclones and flooding. Alongside immediate impacts, disturbance of all kinds may lead to further feedbacks that affect forest functioning (Advance 15). Implications of disturbance for forest landscape recovery remain largely uncertain [68] but where disturbance is excessive, e.g. heavy logging, it can lead to degradation and hence arrested recovery and loss of core ecosystem attributes and functions [81]. To achieve restoration outcomes, FLR decision-making demands understanding of local disturbance regimes to select and design context-specific restoration interventions. Fire management, for example, is integral to FLR but is highly context-dependent and disagreement remains regarding the natural role of fire in forest dynamics [82]. Both positive and negative impacts of fire regimes on FLR exist, creating rare, early successional habitats, while also depleting key patch types and stand functions and structures [83].

Restoration efforts can be inefficient and vulnerable to high rates of mortality where ongoing disturbances have not been addressed as part of the work. Understanding where and how often disturbances occur, and their impact on recovering forests, is essential for informing their management and how, where and when to restore. In this theme issue, new research from Brazil shows that alternative restoration approaches are needed according to the level of disturbance [70]. The theme issue also reveals that forest plantations in Vietnam are more vulnerable to disturbance than natural forests [68]. And lessons learned from Australia and Tanzania show that forest restoration under globally increasing fire risk requires better scientific understanding of natural fire regimes and pre-fire conditions [83] and that local fire risk is crucial to consider in planning for restoration management costs [32].

#### (ii) Advance 9. How do soil and belowground processes recover from disturbance, and how should they be managed?

Ecosystem disturbance can impact soil through agricultural chemicals, machinery compaction, erosion and, in the most extreme circumstances (e.g. mining), total removal or contamination [84,85]. Accordingly, stabilization of soil and maintaining or enhancing hydrological functions and quality are frequently cited within forest restoration objectives. Soils and belowground processes are under-represented in the restoration science literature, with relatively limited knowledge on the impacts of disturbance, or the role of plant–soil interactions in ecosystem recovery. In disturbed or cleared forests, biogeochemical cycling is likely to be disrupted if belowground symbiont or faunal communities are altered, because of their links to aboveground vegetation performance [86–88]. Mycorrhizal inoculations can thus be incorporated with plantings to facilitate establishment, growth and canopy closure [89,90], with outcomes depending upon plant functional type, restoration context and time [91]. However, the role of belowground diversity can be complex, and data are lacking to identify levels of degradation that would necessitate the use of soil treatments [92,93]. There is also still substantial uncertainty and inter-site variability in soil carbon accumulation rates under forest recovery [94]. Science advances are therefore needed to understand whole-ecosystem carbon fluxes under restoration, to avoid inadvertent carbon losses from soil.

Context-specific guidelines are mostly lacking for maximizing restoration success on heavily degraded soils. This is despite degraded soils presenting the greatest ecological challenge for forest restoration, requiring the highest cost and level of management input [95]. In this theme issue, a review of tree-planting approaches indicates that the plant–soil interface should be considered when selecting species for planting; survival may be enhanced by species with key functional traits [96]. For example, and also in this theme issue, Werden *et al*. [97] demonstrate that root traits of planted trees can be used to predict species success in dry forests. Further new research in this theme issue shows how information on soils is necessary for tailoring restoration and remediation methods to local site conditions [70,98].

#### (iii) Advance 10. How does forest restoration impact water quantity and quality?

Uncertainties about the hydrological impact of forests and forest management go back more than a century [99]. These mostly relate to impacts on streamflow parameters, but there are also many misconceptions about the relationships between forests and water [100]. These misconceptions frequently find their way into public discourse and policy, most notably that restoring forests leads to an increase in stream flow [101]. Forests have deeper roots and higher leaf area index than other vegetation types and hence have higher rates of evapotranspiration. Therefore, as forest restoration progresses and plant biomass increases, water use is expected to increase, reducing flow into waterways and groundwater, and thus also total catchment-scale water yield [102,103], potentially resulting in downstream water conflict [104]. It is also frequently misconceived that flooding is best reduced by large-scale reforestation [105,106] and the protective role of forest cover against downstream floods may be overestimated [107]. Empirical evidence shows that a well-developed forest can mitigate small-to-medium flood peaks, but that the type of vegetation cover is irrelevant for large flood events [106].

One of the most important contributions that forest restoration can make to the hydrological functioning of catchments is the reduction in surface soil erosion and the delivery of high-quality water. This can be achieved by managing catchments to retain a cover of low vegetation and leaf litter and minimizing soil disturbance, particularly along stream banks [108]. While much is known about hydrological processes and their relationship with forests, there is a tendency to make generalizations that are frequently inappropriate or misleading and to rely on simple cause–effect relationships. Natural environments are extremely complex, however, and scientific advances are needed to understand how landscape hydrology is affected at each stage of the forest restoration process. Some remaining knowledge gaps are due to complexities and dynamic interactions among soil, biological and hydrological systems and the difficulties of quantifying the impact of restoration on hydrological outcomes under different site conditions [109]. There are also substantial knowledge gaps in the socio-economic consequences of unintended changes in water yields to local and downstream communities [101,106].

A considerable amount of forest hydrology research has been carried out at the scale of hillside plots (as opposed to catchments). Such studies are very useful in exploring hydrological processes (such as infiltration, overland flow, sediment transport and subsurface water flow) and local-level interactions among climate, soil, vegetation, topography and management practices [110]. However, plot-level parameters measured *in situ* cannot be expected to produce accurate predictions at all scales [111], and there are uncertainties when extrapolating findings from plot to catchment scales, or from small to large catchments [112]. The link between plot-scale and catchment-scale outcomes is critical for hydrological outcomes to be fully considered in landscape-level forest restoration planning.

#### (iv) Advance 11. What are the roles and impacts of animals and how should they be managed?

Vertebrate and invertebrate animals are crucial to restoration success or failure. They can eat or trample regrowing plants but can also be crucial for stimulating recovery, particularly at forest edges [113], contributing to nutrient cycling and assisting with seed dispersal, pest predation, pollination and hence also natural regeneration. Local animal extinctions can lead to loss of ecosystem functions such as dispersal [114] or trophic down-control of herbivory [115], reducing the overall resilience of the forest (socio-)ecological system [116]. Thus, during restoration, a focus on strategies that can facilitate recovery of seed dispersal functions in a degraded landscape may be of particular benefit. Seed dispersal in the tropics is often animal-controlled and can facilitate maintenance of forest plant diversity and acceleration of tree species community turnover [117]. Large mammals are important dispersers of large-seeded tree species [119] and can assist natural forest regeneration pathways [120]. These faunal impacts on forest restoration have rarely been quantified, making understanding mechanisms of faunal influence and their importance challenging.

Dispersal constraints and connectivity of habitats for animals are also important research and knowledge gaps. Targeting data collection (e.g. movement data and gut passage times data) and analysis (network analyses and agent modelling) will assist in developing research tools and a framework for their use in FLR planning. Information on the relative importance of spatial, environmental and intrinsic factors acting as dispersal constraints is essential for connectivity modelling techniques which may assist in quantifying habitat connectivity for key species involved in seed dispersal networks [121]. Further, data on landscape and life-history attributes, as well as interactions between animals and tree species and seeds, will be important to develop and parameterize individual-based models that can reconstruct animal movement through the landscape. Lastly, network-level metrics characterizing interaction diversity and specialization will be vital to identify patterns of ecological community assembly and species with key roles within the network using seed dispersal network analysis [122].

In this theme issue, we see two quite contrasting faunal considerations for landscape restoration. New data from Panama show that the diversity and types of animals able to disperse seeds is dependent upon proximity to remnant forest, limited hunting and successfully advancing forest succession [117], while in Tanzania forest restoration for enhancing landscape connectivity is predicted to increase elephant activity on farms and hence also human–wildlife conflict [31].

#### (v) Advance 12. How can trees be planted to recover native forests and achieve multiple objectives?

**Figure 3.**
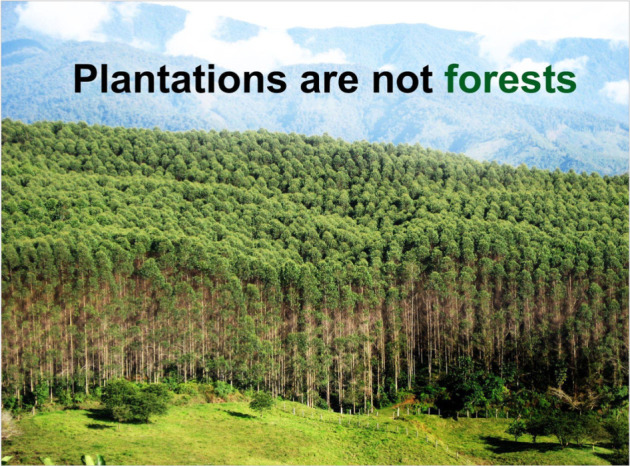
‘Plantations are not forests!’ has been a common slogan in campaigns led by conservation NGOs. This statement derives from a combination of interconnected factors relevant to most of our 15 essential advances, e.g. the use of ecologically inappropriate tree species (poor adaptation to local conditions, invasives, etc.), land-grabbing, loss of food security and limited benefits for local people, who have often been inappropriately displaced from plantation regions. Reproduced with permission from Friends of the Earth International. (Online version in colour.)

Tree-planting is central to most forest restoration projects and yet it remains controversial because of multiple past and ongoing widespread and large-scale mistakes and misclassifications, e.g. planting monocultures [123], exotic/invasive species [124] or ecologically inappropriate species [125], inappropriate locations [126] and inadequate local input/collaboration [49]. Decisions regarding where to plant trees remain challenging, in terms of both where to focus restoration in a landscape (Advance 2), and what species and spatial arrangements to use for planting to maximize potential for natural regeneration and to minimize costs [127]. Consequently, restoration plantings often have high rates of mortality, inadequate species composition and hence also low functional or socio-economic value [25]. Unclear and inconsistent definitions of forest have led to misunderstanding and confusion [128] and even alleged exploitation for political means or profit, prompting multiple campaigns to promote natural forests (figure 3).

Under FLR, a widely adopted framework indicates that the definition of forest must include ecosystem services, value to local livelihoods, biodiversity status and connectivity, all relative to pre-existing forest on the same land [128]. Likewise, reforestation schemes must aim to achieve both ecosystem function and local value at landscape scale. However, the most appropriate species from an ecological perspective can be entirely contrary to those from a socio-economic perspective [129]. And yet, even relatively established procedures for species selection do not include steps to help reconcile these contrasts, e.g. through engagement with all local stakeholders [96]. To feed into this, more research is needed on species–site matching, considering social and biophysical factors, across a broader range of sites and on planting procedures for ecosystem functioning and restoration outcomes. Emerging tools may help to broaden species selection, e.g. Diversity for Restoration, D4R [130], and would benefit from further trials.

In this theme issue, a critique of the widely employed Framework Species Approach shows that procedures for selecting species are inconsistent and ill-defined [96]. Accordingly, new data in the theme issue show that plantings are often species-poor [69], which may be partly due to limited infrastructure to access local seed sources or limited knowledge of nursery practices for a broad set of species. The same study also shows that planting success varied with prior land use, which may alter species suitability. Forest plantations with multiple species are likely more successful than monocultures [131] and have greater natural resistance to pests and disease [132]. Exotic species are also still often used, e.g. as nurse trees to encourage shade for natives [133] or to fix soil nitrogen [95], but further research in this theme issue suggests that exotic tree presence reduces overall biodiversity [134]. In order for tree-planting to be successful, other research in the theme issue highlights important decisions regarding correct selection of species (for drought tolerance; [97]) and planting location (for maximizing survival and natural succession; [70]).

#### (vi) Advance 13. What are the impacts of native and exotic competitive plants and how should they be managed?

Light-loving plants, e.g. vines, shrubs, ferns, bamboo, bracken and grasses, grow rapidly following heavy forest disturbance worldwide and compete with juvenile trees for sunlight, nutrients and resources [135]. These competitive plants can potentially delay or prevent forest recovery, affecting tree diversity, biomass, structure and function [136]. In the light of the global increase in prevalence and severity of disturbance (Advance 8), competition from these plants will increase. Thus, determining where, when and how they can be best managed will be crucial for successful FLR.

The prevalence of competitive plants in dry, fire-dominated landscapes poses a significant challenge for forest restoration. Worldwide, grasses can fuel wildfires, change the nature of mammalian herbivory, and have been implicated in tipping points between forest and savannah [137–139]. In moist and wet forests, lianas and other vines are affecting the recovery of disturbed forests on a pantropical scale, with growing evidence suggesting a second tipping point in forest recovery [140]. However, there is debate regarding the benefit of temporary removal by cutting to stimulate tree growth [141], including potentially negative impacts for biodiversity, drought and protective ‘bandage effects’ on regrowing trees [118,140]. Knowledge is even more limited for other shrubby and herbaceous plants. For example, bracken ferns affect habitat recovery on every continent except Antarctica [142,143], and bamboo can supplement or supplant the effects of lianas on woody regrowth [144].

If potential impacts of competitive plants can be adequately understood and predicted, restoration methods can be selected that are more appropriate and cost-effective (Advance 16). Competitive plant removal is a standard treatment alongside tree-planting, as used successfully in Costa Rica in this theme issue [127]. Thresholds of forest biomass are also used in the theme issue to demonstrate widespread potential for competitive grass, bracken and liana management across a region of high biodiversity value in Tanzania [32]. However, we also see in the theme issue that for other regions, e.g. Southeast Asia, information on competitive thresholds is too limited to draw conclusions for management [69].

#### (vii) Advance 14. How does success vary with restoration method and when should each be applied?

For recovering the species composition and function of native forests, restoration methods should be selected that best facilitate the natural process of succession [11]. Tree-planting is the most widely known, and financed, forest restoration tool (Advance 13) but is often used when ecosystems would recover naturally with a less intensive approach [145]. Tree-planting is more costly than most other restoration methods and, when applied unnecessarily, can have serious consequences for recovering biodiversity, stem survival and ecosystem function [11]. These costly mistakes can jeopardize project success because restoration method selection must instead consider multiple factors [146], e.g. target ecosystem, degradation level, distance to source populations, local knowledge, climate, access and labour.

Restoration practitioners have long appreciated that increased degradation requires more intensive restoration methods, and hence also more time and resources [147]. For example, Elliott *et al*. [95] present a general framework for selecting methods based on five stages of degradation. Alternative approaches should be considered as ways to supplant, support and enhance the success of tree-planting depending on context-specific needs. However, the various methods for restoring native forests, e.g. tree-planting versus assisted and natural regeneration, have not been rigorously compared in most tropical regions [148]. Assisted natural regeneration techniques have been used for many decades to bring about more rapid recovery of valuable timber species [149], e.g. removal of competing plants through weeding and climber-cutting (Advance 15) and through liberation thinning, i.e. removal of non-preferable or pioneer species to benefit later successional species. Enrichment planting, a specific form of tree-planting, can supplement these approaches to expedite canopy closure and the recovery of absent, rare or poorly recruiting species [150,151].

Practitioners require better understanding of when low-cost interventions will suffice and even out-perform high-cost restoration approaches, e.g. through investigation into critical intervention points to define when alternative restoration methods should be applied. In this theme issue, a review of restoration methods emphasizes that more costly approaches can be justified when sufficient value or other benefits are accrued [96]. Banin *et al*. [69] show that active restoration tends to increase rates of basal area accumulation, relative to naturally regenerating forests in Asian forests, but the effect is variable across landscapes. More knowledge is also needed about the implications of landscape mosaics for selecting restoration methods, to ensure that FLR landscapes are not managed with broad-brush approaches. In this theme issue, Wills *et al*. [32] use a combination of local expert knowledge to identify approximate levels of biomass loss expected to result in more intensive management approaches, emphasizing that restoration methods are not expected to be uniform across landscapes.

#### (viii) Advance 15. How should progress be measured and monitored?

Monitoring outcomes of restoration interventions provides the basis for adaptive management and reassessing project goals and methods. Monitoring is also a highly effective way to engage local communities, through participatory activities, selection of indicators and regular visits to restoration sites [152]. As regular designers, users and reviewers of scientific method, scientists are well-placed to facilitate data collection for monitoring, and to convey these methods and findings to practitioners and other stakeholders. And yet, in this theme issue, we see that restoration scientists are not always succeeding in their choice of method, reporting and focus [153] or taking approaches that facilitate effective communication to stakeholders [13]. For scientific measurement and monitoring to appeal to all stakeholders more directly, and account for both environmental and socio-economic priorities, indicator selection needs to consider the full range of FLR project goals, e.g. using the FAO/WRI AURORA approach [154]. Furthermore, with the increasing complexity of restoration science, and emerging technologies for surveying and sampling, scientists, now more than ever, need to ensure that their proposed measures and methods for monitoring are realistic and standardized for use by practitioners.

In the early stages of FLR projects, baseline data are needed, both to make evidence-based decisions near the outset and to evaluate outcomes going forward. Yet, baseline conditions are often not measured, with both species and structural indicators of ecosystem recovery often instead compared with ‘undisturbed’ reference sites, which themselves are also often missing from the landscape, or simply not measured. Most knowledge regarding restoration outcomes is also only based on small, localized experimental plots, limiting potential to guide projects at larger scales [155]. This absence of a realistic benchmark represents a lack of untreated control sites, reducing the ability to identify effects of landscape and geomorphic factors on restoration outcomes [156]. Indicators are also typically too few to fully understand ecosystem complexity and lacking measures of broader ecosystem function (e.g. productivity, soil health and functional diversity) [157,158]. Science and monitoring have also so far rarely measured, monitored and provided solutions to solve leakage of negative impacts on forests outside of sites targeted by restoration or conservation [159].

Ideally, a combination of leading and lagging indicators should be applied to monitor restoration progress [160]. Most restoration projects rely only on lagging indicators, which are effectively measures of efforts taken rather than actual outcomes. For example, statements such as ‘1000 trees planted’ or ‘20 hectares restored’ fail to provide information about how social or environmental conditions were improved by interventions. Leading indicators, in contrast, can be important predictors of later restoration success, e.g. in Costa Rica, two site variables were reasonable predictors of natural forest recovery after 8.5 years [161]. Socio-economic data perhaps have the greatest emerging potential as leading indicators and yet have been traditionally overlooked by restoration ecologists [158]. Thus, the measurement of restoration progress could include human wellbeing indicators such as household assets, health, social relations, forest access, education, security, involvement in decision making and livelihood satisfaction [162]. Adopting shared social and ecological indicators of restoration progress and outcomes permits broader assessments and more rigorous comparisons of the effects of different approaches and contexts and adherence to the holistic principle of FLR. This is now possible owing to the development of the Restoration Project Information Sharing Framework [163].

Longitudinal studies lasting morer than 5 years are rare among the published restoration literature [69] but are urgently needed to understand the mechanisms, ecosystem service flow and other long-term outcomes of forest restoration and management [155]. Assessing recovery rates using chronosequences does not offer the same level of information provided by long-term studies and can be misleading [164]. Long-term monitoring within individual restoration sites provides critical information on demographic rates of seedlings, saplings and trees which inform the mechanisms of treatment effects [127]. Large, long-term datasets are also crucial for proving and recognizing tipping points, which can require very large amounts of data [165]. These crucial moments in time can be essential for FLR decision making, indicating critical intervention points for action before ecosystem or social resilience is lost, e.g. for selecting restoration locations and methods (Advances 7, 13, 15 and 16) and for identifying critical deficiencies in wellbeing or ecosystem services (Advance 2). Similarly, managers need to also identify signs that management intervention is not required, hence directing time and resources towards more appropriate activities or locations.

Measurement and monitoring are central to all articles in this theme issue. Monitoring data from Ecuador and Southeast Asia, respectively, show that ecological monitoring has potential for maximizing restoration outcomes for plant survival and growth [69] and for seed dispersal [118]]. We also see that indicators of forest structure or biomass are less sensitive to sampling area than are indicators of tree species diversity and composition, but small sample plots require standardizing for sample coverage when comparing species diversity [166]. Various articles in the theme issue also emphasize the importance of socio-economic measurement and monitoring, for pre-assessment of community restoration potential [47], evaluating human–wildlife conflict [31] and ensuring human wellbeing benefits [45].

## 3. Conclusion

The extensive knowledge gaps that we have identified indicate that scientific advances are urgently needed to inform FLR interventions. As restoration is poised to ramp up, we need to ensure resources used lead to fruitful outcomes and avoid costly and demotivating mistakes and loss of trust among actors. We have shown that scientific advancements should be realistic, at relevant scales and data-driven, often requiring complex, spatially explicit, interdisciplinary approaches to account for typical landscape diversity, multiple objectives, drivers and trade-offs. There is still a lot to learn regarding disturbance, soil, secondary vegetation, animals and the restoration methods for managing these while also encouraging natural processes. There is also still a lot to learn regarding socio-economic and governance challenges, and methods for assessing these. While the articles in the Theme Issue have made advances in all these biological and socio-political areas, they have not addressed restoration uncertainties relating to hydrology (table 1; Advance 10), suggesting that this is an especially significant gap. Furthermore, as recently observed for restoration practice [11], the greatest challenges for science appear to lie in the conceptualizing, planning and assessment stages of restoration, thus identifying a need to develop an evidence base for why, where and how to restore. Therefore, like indicators of success, science should aim to provide evidence that leads, not lags behind, restoration practice.

Without specifically targeting tropical regions, most of the articles in this theme issue have tropical focus, and all of the essential advances have relevance for developing tropical regions. We conclude that the developing tropics remain the priority location for FLR research, which was significantly lacking until recent years [158]. Within these regions of high dependence on forests by local people, land-use priorities could be better identified if scientists and policymakers work with people and elected representatives of these people at local scales. Restoration, like any land-management intervention, must ultimately be implemented by people in their distinct social and ecological contexts. *In situ* scientific research and monitoring provides an excellent opportunity to engage and learn from local people and hence also improve the incorporation of science into practice. Armed with clearly communicated evidence at the outset of restoration planning, donors, decision makers and practitioners should be better placed to develop informed objectives and realistic targets. To facilitate and build from this foundation, scientists also need to form working relationships with other sectors across the whole AMC if their research findings are to be embraced and used. The current theme issue uniquely brings together multiple disciplines to appeal to multiple sectors, and to help connect scientists and practitioners who historically have worked quite separately.

## Data Availability

The data are provided in the electronic supplementary material [167].
